# An Experience with Chloretone in Cholera Morbus

**Published:** 1900-10

**Authors:** Edwin B. Reed

**Affiliations:** Asbury Park, N. J.


### AN EXPERIENCE WITH CHLORETONE IN CHOLERA MORBUS.

BY EDWIN B. REED, M.D.,
ASBURY PARK, N. J.

It was recently my experience to be called in the night to treat a severe case of cholera morbus which taxed my available resources to no small degree, and culminated in an unexpected triumph for that remarkable drug chloretone, anodyne, hypnotic and local anesthetic. I first administered two hypodermatic injections of morphine, of one-fourth grain each, which relieved the pain, but failed to allay the extreme nausea. I then tried successively oxalate of cerium, bismuth subnitrate, cocain, iced brandy, iced champagne, carbolic acid with bismuth, and creosote, but without the slightest improvement. Eventually the symptoms became so alarming that I was obliged to give a hypodermatic injection of strychnine, followed in a little while by one of nitroglycerin. By this time the patient was so utterly exhausted that she fell off into a momentary sleep. At that juncture it occurred to me that if I could induce the desired repose the vomiting would cease, and with that object in view I gave ten grains of chloretone in saturated aqueous solution. In two or three minutes the patient said her stomach felt "cool," and soon the circulation became normal, the color was restored to the pallid countenance, and ere long the woman fell asleep. To my extreme satisfaction the sleep continued calmly for four or five hours, after which improvement rapidly took place.

In my next case of this kind I shall give chloretone before trying anything else.

